# Antistaling properties of encapsulated maltogenic amylase in gluten‐free bread

**DOI:** 10.1002/fsn3.1865

**Published:** 2020-09-20

**Authors:** Sepideh Haghighat‐Kharazi, Mohammad Reza Kasaai, Jafar Mohammadzadeh Milani, Khosro Khajeh

**Affiliations:** ^1^ Department of Food Science and Technology Sari Agricultural Sciences and Natural Resources University Mazandaran Iran; ^2^ Department of Biochemistry Tarbiat Modares University Tehran Iran

**Keywords:** beeswax, encapsulation, gluten‐free bread, maltogenic amylase, staling

## Abstract

Staling of bakery products especially gluten‐free products is a challenge on the development of these products. For retarding staling of gluten‐free bread, maltogenic amylase (MAase) at concentrations of 8.2, 45, and 82 mg/ml was encapsulated into beeswax (BW) at 1%, 2.5%, and 4% levels. Results showed the treatment with 8.2 mg/ml MAase and 2.5% beeswax had the highest encapsulation efficiency (42.04%) and chosen for subsequent experiments. The size of encapsulated particles was 362.70 nm and had a zeta potential of −15.35 mV. Surface morphology of encapsulated MAase was almost spherical with layered appearance. The free and encapsulated MAase with the activity of 5.2 µmol/min were used in gluten‐free batter and breads, respectively. In the rheological tests, batters containing free and encapsulated MAase showed lower cross over point than control batter (without enzyme or wall material) (59 and 53 Hz, respectively). Encapsulated MAase contained bread had darker crust, whiter and softer crumb, and more aerated structure in comparison with free MAase loaded one. Both breads containing MAase as free or encapsulated had higher moisture content and water activity in crust and crumb than control bread. However, bread with free MAase had softer crumb after four days of storage, and bread with encapsulated MAase had higher sensorial acceptability than other breads after 2 and 4 days of storage.

## Graphical abstract

For retarding staling of the gluten‐free bread, maltogenic amylase (MAase) at concentrations of 8.2, 45, and 82 mg/ml was encapsulated into beeswax (BW) at 1, 2.5, and 4% levels, among which the treatment with 8.2 mg/ml MAase and 2.5% beeswax showed the highest encapsulation efficiency (42.04%), thus chosen for the subsequent experiments. The free and encapsulated MAase with the activity of 5.2 µmol/min were used in the gluten‐free batter and bread. In the rheological tests, batters containing free and encapsulated MAase showed lower crossover point than the control one (without enzyme or wall material). Both breads containing MAase, as free, or encapsulated had higher moisture content and water activity in crust and crumb than the control bread, but bread with encapsulated MAase had softer crumb after two days of storage, and its sensorial acceptability was higher than other breads during storage.

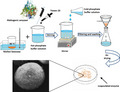

## 1 INTRODUCTION

A persistent perturbation of small intestine, celiac disease, is due to ingestion of gluten proteins of wheat, barley, rye, triticale, and possibly oats (Moore, Dal Bello, & Arendt, 2008). Cure of this food intolerance is to follow a strict gluten‐free diet for whole life of the patient (Demirkesen, Mert, Sumnu, & Sahin, 2010). However, gluten removal leads to many qualitative deficiencies such as low volume, dry texture, poor flavor, and shorter shelf life of gluten‐free bread in comparison with wheat bread (Demirkesen, Sumnu, & Sahin, 2013; Rostamian, Milani, & Maleki, 2014).

For retarding staling, MAase is highly used in the baking industry (Li, Wang, Park, Gu, & Li, 2018). It has been appeared more as an exo‐acting amylase than an endo‐acting enzyme at a superior temperature (Gomes‐Ruffi, da Cunha, Almeida, Chang, & Steel, 2012). Unlike α‐amylase, MAase due to its inactivation during baking is not able to further hydrolysis of starch and producing soluble dextrins, which is the cause of gumminess in prepared bread (Gerrard, Every, Sutton, & Gilpin, 1997). In comparison with enzymes, MAase is unique in yielding significant softness to bread and maintaining a high level of crumb elasticity during storage. It is extensively used in the baking industry to retard bread staling (Gomes‐Ruffi et al., 2012; Li et al., 2018).

Lipid‐based wall materials in enzyme encapsulation for application in the baking process have been done by some scientists. Dusterhoft et al. (2006) have reported the encapsulation of α‐amylase into hydrogenated stearin fraction of palm kernel oil by spray chilling technique for application in bread formulation. Plijter and Meesters (2000) also investigated the application of coated lipase into maltodextrin and other materials on the quality of baked products. Waxes have good stability at various moisture content and pH. They have no immunogenicity for human because of their insoluble nature. Moreover, producing technique of waxes microparticles does not need complex devices, organic solvents, and heating for a prolonged time (Hassan, Eshra, & Nada, 1995; Ranjha, Khan, & Naseem, 2010). Beeswax is a permitted additive used in the European Union (E901‐903) and is frequently used for encapsulation of drugs and bioactive compounds (Chitprasert & Sutaphanit, 2014; Mellema, Van Benthum, Boer, Von Harras, & Visser, 2006; Ranjha et al., 2010). In our previous work, we have optimized the encapsulation of α‐amylase into beeswax using RSM and applied the optimized one into the gluten‐free batter and bread (Haghighat‐Kharazi, Milani, Kasaai, & Khajeh, 2018). Here in this work, we aimed to investigate the application of beeswax for encapsulating MAase and its application into gluten‐free batter and bread. After determination of efficiency and physicochemical characteristics of encapsulated MAase, we studied the application of encapsulated enzyme into gluten‐free rice‐based batter and bread and assayed the qualitative, sensorial, and staling features of prepared batter and bread.

## 2 MATERIALS AND METHODS

### 2.1 Materials

Maltogenic amylase (MAase) (EC 3.2.1.133) from *Bacillus stearothermophilus*, soluble starch, and 3, 5 dinitrosalicylic acid (DNS) were purchased from Sigma (St. Louis, Mo, USA). White beeswax was purchased from Samchun Pure Chemical Co. (South Korea). Sodium dihydrogen phosphate dihydrate, sodium hydroxide, and potassium sodium tartrate were obtained as analytical grades (Merck, Germany). Rice flour from *Behnam* variety (92.71% dry matter; 11.87% protein, 2.76% fat; 0.28% ash) was acquired from a local farmer (Mazandaran, Iran). Chickpea flour (Seity, Iran); diacetyl tartaric acid esters of monoglycerides (DATEM) (Pars Behbood Asia, Iran); hydroxypropyl methylcellulose (HPMC) (Fluka, Switzerland), instant yeast (Razavi Yeast, Iran), vegetable oil (Behpak Industrial company, Iran), salt, and sugar were also used for the preparation of gluten‐free batter and bread.

### 2.2 Encapsulation of MAase into beeswax

The encapsulation of MAase into beeswax was done using the method described by (Haghighat‐Kharazi et al., 2018; Kheradmandnia, Vasheghani‐Farahani, Nosrati, & Atyabi, 2010) with minor modifications. In this method, encapsulated MAase was prepared by emulsion‐congealing technique. Beeswax was melted in a water bath at 90°C. Then, the melted beeswax (1, 2.5, or 4 g) was poured into 100 ml of stirring phosphate buffer solution (50 mM, pH 7), which had been previously heated to a temperature higher than the melting point of beeswax (˃+5°C). Next, Tween 20 and MAase (8.2, 45, or 82 mg/ml) were added to the molten lipid and buffer mixture. After maintaining the stirring for 2 min using a magnetic stirrer (Heidolph, Kelheim, Germany), the emulsion was immediately cooled down using the same volume of cold phosphate buffer solution (0–2°C) under mechanical stirring to produce spherical solid particles. Finally, the obtained solid spheres were collected and filtered through Whatman no.3 filter paper and rinsed by distilled water to remove any surfactant and enzyme residues. The air‐drying process was performed at room temperature (25°C) for 24 hr to produce single and free‐flowing solid spheres. Final products were stored at 4°C for further experiments.

### 2.3 Determination of enzyme encapsulation efficiency

The efficiency of MAase encapsulation was evaluated by determining the amylolytic activity of free and encapsulated enzymes. MAase activity was assayed according to the method of Bernfeld (1955) using spectrophotometer (Perkin‐Elmer, Lambda 25, UV‐vis) at 540 nm for determining the amount of produced maltose. One unit of α‐amylase is expressed as the amount of enzyme producing 1 μmole of maltose per min. To determine the encapsulation efficiency, bellow equation was used (Amid, Manap, & Zohdi, 2014):

(1) $$\text{Encapsulation Efficiency (}\%)=\frac{\text{specific activity of encapsulated enzyme}}{\text{specificactivity of free enzyme}}\times 100.$$

The encapsulation efficiency of various formulations is presented in Table 1. The highest encapsulation efficiency (42.04%) was attributed to the treatment code of BW_4_‐MAase (beeswax = 2.5% and enzyme concentration = 8.2 mg ml^−1^) as the optimized one.

**Table 1:** Effect of beeswax and MAase concentrations on encapsulation efficiency of prepared particles

Treatment code	Independent variables		Encapsulation efficiency (%)
	Beeswax concentration (%)	Enzyme concentration (mg/ml)	
BW_1_‐MAase	1.00	8.20	15.72 ± 0.41^bc^
BW_2_‐MAase	1.00	45.00	0.35 ± 0.30^e^
BW_3_‐MAase	1.00	82.00	1.01 ± 0.17^e^
BW_4_‐MAase	2.50	8.20	42.04 ± 5.37^a^
BW_5_‐MAase	2.50	45.00	1.99 ± 0.90^de^
BW_6_‐MAase	2.50	82.00	1.14 ± 0.61^e^
BW_7_‐MAase	4.00	8.20	21.51 ± 12.29^b^
BW_8_‐MAase	4.00	45.00	10.47 ± 4.79^cd^
BW_9_‐MAase	4.00	82.00	4.03 ± 2.32^de^

Note

BW‐MAase = Encapsulated MAase into beeswax. The values are expressed as Mean ± Standard deviation. Means with superscripts of different letters are significantly different (*p* < .05).

### 2.4 Particle size and zeta potential measurements

Particle size and zeta potential of encapsulated enzyme were estimated by a dynamic light scattering technique using a Malvern Zetasizer Nano‐Series (Nano‐ZS, Malvern Panalytical Instrument, England).

### 2.5 Scanning electron microscopy (*SEM*)

The morphology of the encapsulated enzyme was determined by *SEM* instrument (FE‐*SEM*, Hitachi S4160, Japan), under an acceleration voltage of 20 kV. The microstructure of bread was also determined using *SEM* (Electric Systems, Cambridge, Model 1455VP, UK). The bread samples were air‐dried. Each sample of encapsulated enzymes or bread was placed on a copper grid and was coated with a thin layer of gold.

### 2.6 Preparation of gluten‐free batter and bread

Gluten‐free batter and bread were made according to the method published recently (Haghighat‐Kharazi, Jafar, Kasaai, & Khajeh, 2019; Haghighat‐Kharazi, Kasaai, Milani, & Khajeh, 2020; Haghighat‐Kharazi et al., 2018). The ingredients were based on 100 g of flour (rice: chickpea flour ratio, 80:20), and 125 ml of water (30°C). The other ingredients except flour and water were 5% sugar, 2% salt, 6% vegetable oil, 1% HPMC, 0.5% DATEM, 3% instant yeast, MAase or free enzyme with the activity of 5.2 µmol/min, and wax 0.88%. Dry components were mixed together. Water and oil were added to the dry components and mixed at speed 3 using a hand mixer (Black & Decker, model M220, USA) for 4 min. Then, the batter was poured into a mold and fermented for 30 min (35°C, 80% humidity). Finally, batter was baked for 25 min at 220°C in a semi‐industrial electrical oven (Mashhad Baking Industries Co., Iran). After baking, they were cooled down to ambient temperature and stored in the polyethylene bags. Four different breads containing BW‐MAase (bread containing encapsulated MAase into BW), BW (bread containing BW), MAase (bread containing free enzyme), and control (bread without enzyme or BW) were prepared. Ten loaves of each bread were made for each treatment.

#### 2.6.1 Rheological properties of gluten‐free batters

The rheological properties of gluten‐free batters were measured using a rheometer (MCR 301, Anton Paar GmbH, Germany) equipped with parallel plate geometry (50 mm diameter, gap 1 mm) (Haghighat‐Kharazi et al., 2018). The experiments were performed as follows: (1) an amplitude sweep to determine the limit of the linear viscoelastic region of batters at strain of 0.001%–100% and an angular frequency of 1 Hz (data were not shown). The following experiments were performed using the information derived from this linear viscoelastic region; (2) a frequency sweep (0.1–100 Hz) with a target strain of 1% at 25°C to determine storage modulus for evaluation of elastic response, G′, and loss modulus for estimation of viscous behavior, G″; and (3) a temperature sweep from 20 to 90°C at a heating rate of 4°C/min and an angular frequency of 1 Hz and a strain of 0.05%, to determine the effect of temperature on the structure of batters, complex modulus, G*, versus temperature.

#### 2.6.2 Physicochemical parameters of bread

Weight loss percent (WL%) during baking was measured as described by Demirkesen et al. (2013). Loaf specific volume was evaluated using the rapeseed displacement as described in the approved method of AACC, 10–05 (AACC, 2000). Volume, symmetry, and uniformity indexes were measured as described in AACC, 10–91 (AACC, 2000). Crumb/crust weight ratio was calculated based on the weight of crumb to the weight of crust (Curic et al., 2008). Crumb porosity of loaves was assayed using a flatbed scanner (Model Scan jet 2410, HP, Cupertino, USA) with a resolution of 300 dpi and the data were processed using an Image‐Pro plus 4.5 (Media Cybernetics Inc., USA) (Esteller, Zancanaro, Palmeira, & da Silva Lannes, 2006). The crumb porosity features chosen were mean cell area (mm^2^), mean diameter (mm), minimum diameter (mm), maximum diameter (mm), and nonuniformity of gas cells.

#### 2.6.3 Estimation of crumb and crust color

The color of crumb and crust were estimated using CIE *L*, *a*, and *b* color scale, a Minolta Chromo‐meter (CR‐100); and a tristimulus color analyzer (Konica Minolta Sensing, Inc., Sakai, Osaka, Japan). The values of CIE *L*, *a*, *b* and whiteness (100 ‐ [(100‐*L*)^2^ + (*a*)^2^ + (*b*)^2^]^1/2^) were recorded for each bread loaf [20, 21].

#### 2.6.4 Texture analysis

Texture profile analysis (TPA) of samples was carried out using a texture analyzer (Texture Pro. CT V1.6 Build, Brookfield Engineering Labs, Middleboro, MA, USA) equipped with a 10 kg load cell and a TA25/1000 probe (AACC, 2008). The bread loaves were cut into 25 mm pieces and compressed at a test speed of 1 mm/s with a trigger load of 5 g for compressing the center of the bread to 40% of its original height. Hardness, resilience, cohesiveness, springiness, gumminess, and chewiness were calculated using Texture Expert software (Texture Pro CTV1.6 Build 26). Texture analyses were conducted 2 hours after baking.

#### 2.6.5 Evaluation of bread staling

Breads were stored in the sealed polyethylene bags at room temperature (25°C). At one, two, and four days after baking, breads were subjected to the following tests. Crumb and crust moisture contents were determined by an air oven gravimetric method of AACC, 44‐15 (AACC, 2000). Water activity (a_w_) of crumb and crust was measured using LabSwift‐aw (Novasina, Switzerland). The firmness of bread samples was measured using a texture analyzer (Texture Pro. CT V1.6 Build, Brookfield Engineering Labs, Middleboro, MA, USA), equipped with a 10 kg load cell and a TA25/1000 probe according to the AACC, 74‐09 (AACC, 2008).

Sensory evaluation of staling was performed by 5 panelists to evaluate the loaves for overall acceptability at first, second, and fourth days. Acceptability was determined by scoring the samples from 1 to 5 (1 for an unacceptable and 5 for a high satisfactory).

### 2.7 Statistical analysis

All experiments were performed in two replicates. Data were statistically analyzed using SPSS V16.0 for analysis of variance (ANOVA), followed by Duncan's multiple range test (*p* ˂ 0.05) for determining significant differences.

## 3 RESULTS AND DISCUSSION

### 3.1 Encapsulation of MAase into BW

The effects of BW and MAase concentration on the encapsulation efficiency of encapsulated MAase are presented in Table 1. The highest encapsulation efficiency (42.04 ± 5.37%), was observed using the treatment code of BW_4_‐MAase (BW with the concentration of 2.5% and MAase with the lowest concentration (8.2 mg/ml)). By increasing the enzyme concentration at each concentration of BW, the encapsulation efficiency decreased (*p* < .05) and by increasing the concentration of BW at a concentration of 8.2 mg/ml of the enzyme, the encapsulation efficiency increased and then decreased, and at the concentrations of 45 and 82 mg/ml of MAase, the encapsulation efficiency increased. Therefore, the encapsulation efficiency changes as a function of both the BW and enzyme concentrations. Amid, Tan, Mirhosseini, and Ab.Aziz, Ling, (2011) also reported that serine protease concentration and wall material content had significant influence (*p* ˂ .05) on the encapsulation efficiency of the encapsulated serine protease. However, in another study which was done by our group on encapsulation of α‐amylase into BW using response surface methodology (RSM) with assessing independent variables such as enzyme, BW, and surfactant concentrations and also stirring rate, a great increase in the encapsulation efficiency of the encapsulated enzyme was observed, when the concentration of beeswax decreased and enzyme increased (Haghighat‐Kharazi et al., 2018). Therefore, it seems different enzymes and their amino acid subunits determine their tendencies in introducing and being active in the same wall material. In our another work which is about encapsulation of MAase into maltodextrins with different dextrose equivalents (DE 4–7 and 16.5–19.5), results showed that the encapsulation efficiency increased by increasing and decreasing of enzyme and maltodextrin, respectively. According to these results, we can say that the efficiency of enzyme encapsulation into lipidic or carbohydrate‐based wall materials depends on both the kind of enzyme and wall materials.

### 3.2 Particle size and zeta potential characterization

The particle size of the encapsulated MAase into BW was 96.85 µm. The negative zeta potential of −15.35 ± 0.35 mV was obtained for encapsulated MAase into BW. Encapsulation of α‐amylase into BW also yielded in a low negative zeta potential (Haghighat‐Kharazi et al., 2018). Colloidal dispersions with potentials within +30 to −30 mV tend to coagulate, whereas colloids with potentials greater than +30 mV or smaller than −30 mV are electrically stabilized (Honary & Zahir, 2013). Therefore, the aggregation of this encapsulated enzyme in an aqueous medium is expected (Kheradmandnia et al., 2010).

### 3.3 Morphology of encapsulated enzyme

**Figure 1:**
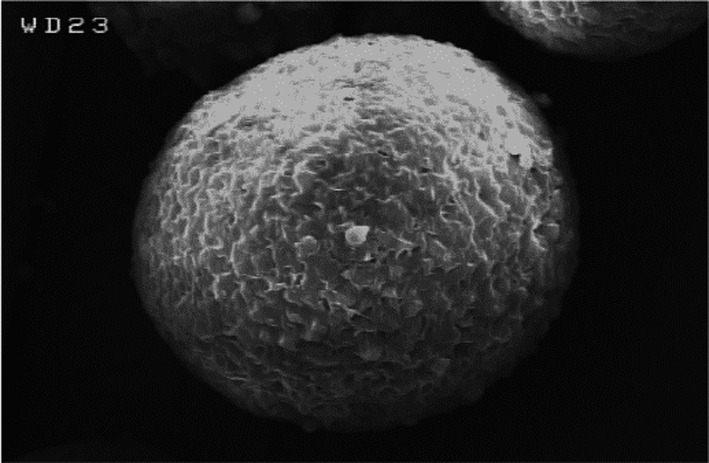
*SEM* micrograph of encapsulated MAase into BW. Magnification was 600×

Surface morphology of encapsulated MAase into BW measured by *SEM* is illustrated in Figure 1. Contrary to surface morphologies of materials obtained from freeze‐drying in matrixes like maltodextrin, which are glassy and an irregular form, surface morphology of encapsulated MAase into BW was almost spherical with layered appearance, which is in agreement with our previous work (Haghighat‐Kharazi et al., 2018; Khazaei, Jafari, Ghorbani, & Kakhki, 2014).

### 3.4 Rheological features of gluten‐free batters

**Figure 2:**
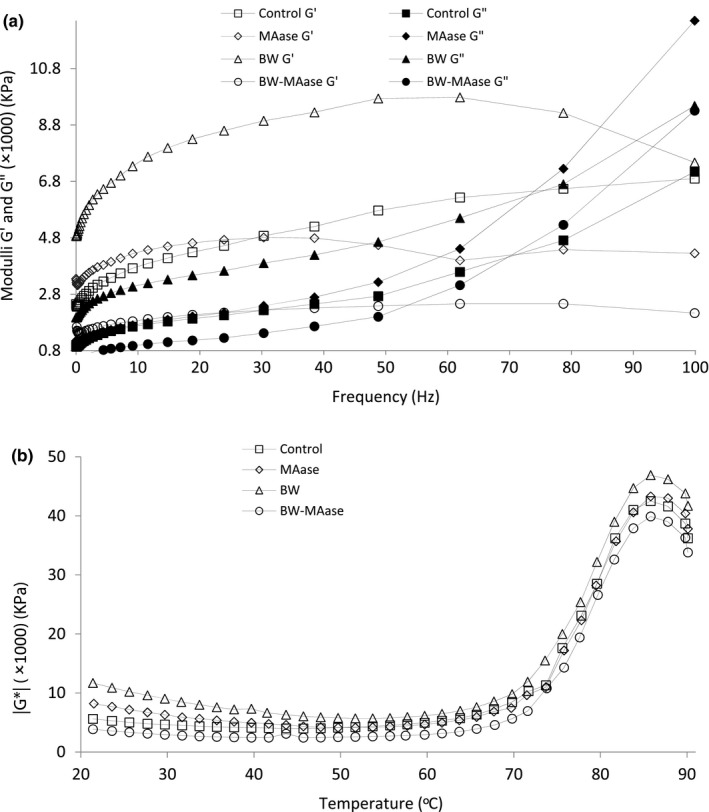
(a) Variation of viscose (G″) and elastic (G′) modulus as a function of frequency; and (b) variation of complex modulus (G*) as a function of temperature. Control (a batter without MAase and BW); MAase (a batter containing free MAase); BW (a batter containing BW); and BW‐MAase (a batter containing encapsulated MAase into BW)

The variations of G′ and G″ as a function of frequency for different gluten‐free batters were illustrated in Figure 2a. The linear viscoelastic region (strain 1%) was identified from the strain sweep experiment (data were not shown). Higher values of G″ (f = 1–100 Hz), and higher values of G′ (f < 30) in comparison with the control were observed for batters except for BW‐MAase batter. The value of G′ (f > 30) has started to drop for all batters except for the control. Similar results for G′ and G″ (f = 0.1–16 Hz), have been reported for gluten‐free batters containing MD (Witczak, Korus, Ziobro, & Juszczak, 2010). The value of G′ decrease with an increase in frequency for the batters containing BW, BW‐MAase, and MAase regarding the control batter. This result indicates that MAase and its encapsulation into BW resulted in some breaks into the system. Dusterhoft et al. (2006) reported that the application of encapsulated amylase into hydrogenated stearin fraction of palm kernel oil in dough caused to a slightly less elastic consistency rather than free amylase. However, MAase has a low activity at low temperatures (<35°C); thus, it may act on damaged starch. Addition of the enzyme would yield in the hydrolysis of α‐(1–4) bond present in starch, leading to the producing of low molecular weight dextrins (Sciarini, Ribotta, Leon, & Pérez, 2012). Therefore, the lower cross over points (which represent changes in batters behavior over range frequency) for MAase and BW‐MAase batters (59 and 53 Hz, respectively) were obtained rather than BW and control batters (90 and 98 Hz, respectively). As beeswax has high hydro repellency nature due to long‐chain fatty acids, a delayed release of the enzyme in the batter is possible (Chitprasert & Sutaphanit, 2014). Some research also proved plate‐like crystals in waxes, organizing the labyrinth effects that were effective in preventing the diffusion of small compounds (Mellema et al., 2006). Nevertheless, in our study, batter contained BW‐MAase showed cross over point in lower frequencies rather than a batter containing MAase.

The effect of temperature on the structure of batters (complex modulus, G*, versus temperature) was illustrated in Figure 2b. Generally, all batters mostly exhibited a similar trend and the same starch gelatinization temperatures.

### 3.5 Physicochemical features of gluten‐free breads

Various physicochemical features of different gluten‐free bread formulations are illustrated in Table 2. All gluten‐free breads showed weight loss during baking, which the lowest was related to the breads containing free and encapsulated MAase (26.24% and 26.97%, respectively) with no significant difference (*p* < .05). Encapsulation of MAase into maltodextrin with DE 4–7 had lower weight loss rather than its encapsulation into BW (24.17% against 26.97%, respectively) (Haghighat‐Kharazi et al., 2019) which could be related to its higher encapsulation efficiency of MAase encapsulated into maltodextrin with DE 4–7 rather than BW (93.35% and 42.04%, respectively). Gluten‐free batters require more hydration than wheat dough to achieve an appropriate consistency. Higher moisture retention (for breads after the baking), yielded in an improvement in the quality of gluten‐free bread via a reduction in firmness (Furlán, Padilla, & Campderrós, 2015). Values of weight loss obtained in this study were higher than the results reported by de la Hera, Martinez, and Gómez (2013) which could be related to a higher water content in our batters formulation (125 g/100 g versus 110 g/100 g of flour), and a higher baking temperature (220°C versus 190°C). The lower weight loss of BW‐MAase bread than BW bread is due to its higher moisture retention, which causes to lower specific volume of BW‐MAase bread (2.58 against 2.78 cm^3^/g) (Table 2). Although the volume index of BW‐MAase bread is higher than BW, this difference like uniformity index was not significant (*p* > .05) (Table 2).

**Table 2:** Physicochemical properties of gluten‐free breads

Parameters	Bread sample			
	Control	MAase	BW	BW‐MAase
Weight loss (%)	27.27 ± 0.42^b^	26.24 ± 0.15^c^	29.98 ± 0.00^a^	26.97 ± 0.46^bc^
Specific volume (cm^3^/g)	2.76 ± 0.10^ab^	2.77 ± 0.09^ab^	2.78 ± 0.00^a^	2.58 ± 0.02^b^
Volume index (cm)	6.30 ± 0.42^a^	5.90 ± 0.28^a^	5.75 ± 1.20^a^	6.25 ± 0.64^a^
Symmetry index (cm)	0.15 ± 0.00^a^	−0.05 ± 0.07^b^	0.25 ± 0.07^a^	0.20 ± 0.00^a^
Uniformity index (cm)	−0.10 ± 0.07^a^	0.05 ± 0.07^a^	−0.05 ± 0.07^a^	0.00 ± 0.28^a^
Crumb/crust ratio	1.75 ± 0.03^a^	1.68 ± 0.09^ab^	1.52 ± 0.08^b^	1.59 ± 0.00^ab^
Mean cell area (mm^2^)	1.44 ± 0.16^a^	0.96 ± 0.37^a^	1.16 ± 0.25^a^	1.19 ± 0.74^a^
Mean diameter (mm)	1.83 ± 0.01^a^	1.82 ± 0.10^a^	1.84 ± 0.04^a^	1.72 ± 0.08^a^
Min diameter (mm)	1.20 ± 0.01^a^	1.19 ± 0.08^a^	1.22 ± 0.00^a^	1.10 ± 0.05^a^
Max diameter (mm)	2.48 ± 0.01^a^	2.45 ± 0.12^a^	2.47 ± 0.05^a^	2.35 ± 0.08^a^
Nonuniformity	1.28 ± 0.01^a^	1.25 ± 0.03^a^	1.25 ± 0.05^a^	1.25 ± 0.04^a^

Note

Control = bread without MAase and BW; MAase = bread containing free MAase; BW = bread containing BW; BW‐MAase = bread containing encapsulated MAase into BW. The values are expressed as Mean ± Standard deviation. Means with superscripts of different letters are significantly different (*p* < .05).

Control bread showed the highest crumb/crust ratio than other breads (Table 2). A low value of this ratio indicates that the incorporation of MAase and BW could result in a thicker crust formation during baking. According to crumb porosity features, breads did not show considerable differences (*p* > .05) (Table 2). However, bread with BW‐MAase formulation showed a lower mean, min and max diameters than MAase and BW breads, which lead to higher mean cell area of BW‐MAase formulation rather than MAase and BW breads (1.19 against 0.96 and 1.16 mm^2^, respectively) with the same nonuniformity (1.25). The higher mean cell area of control bread is likely because of higher diameters of gas cells that also resulted in higher nonuniformity (1.28) than other breads (1.25) (Table 2). A low nonuniformity would lead to a lower compact structure of gluten‐free bread due to a better maintenance of CO_2_ upon fermentation (Arendt, Morrissey, Moore, & Dal Bello, 2008). Costumers usually accept breads with a lower nonuniformity and a high number of medium‐sized average pore (Phimolsiripol, Mukprasirt, & Schoenlechner, 2012).

### 3.6 Color of gluten‐free breads

Color is a significant property of breads, which is related to their texture and aroma, and consumer preference (Esteller & Lannes, 2008). However, in most cases of crust color parameters in Table 3, there were no significant differences (*p* > .05). Application of BW‐MAase in gluten‐free bread formulation resulted in a darker color for the crust (lower values of crust *L* and whiteness) than MAase and BW breads. It seems in this formulation more sugars in the batter are available, which are susceptible for Maillard and caramelization reactions and result in brown pigments which higher values of *a* also confirmed it. However, in the case of free enzyme loaded bread (MAase) with the same enzyme concentration, it showed a little different. Breads with encapsulated MAase into maltodextrins formulations in previous work (Haghighat‐kharazi et al., 2019) also showed the lowest values of crust *L* and whiteness. Regarded to crumb color parameters, again BW‐MAase bread showed lighter and whither crumb than other breads. Negative values for *a* were obtained for all gluten‐free breads; that is, no red color for crumb was observed. Crumb yellowness was also observed for all breads in comparison with control (Table 3).

**Table 3:** Color and textural features of gluten‐free breads

Parameters	Bread sample			
	Control	MAase	BW	BW‐MAase
Crust L	56.41 ± 0.22^a^	60.79 ± 1.44^a^	57.91 ± 1.31^a^	56.91 ± 2.91^a^
Crust a	15.50 ± 0.08^ab^	11.54 ± 1.46^b^	15.32 ± 0.99^ab^	17.46 ± 2.31^a^
Crust b	42.46 ± 0.10^b^	46.41 ± 0.42^a^	46.09 ± 0.51^a^	46.15 ± 1.28^a^
Crust whiteness	36.95 ± 0.28^a^	38.00 ± 1.48^a^	35.55 ± 1.44^a^	34.26 ± 1.64^a^
Crumb L	76.53 ± 1.04^ab^	76.31 ± 0.65^ab^	75.99 ± 0.65^b^	78.68 ± 1.00^a^
Crumb a	−0.96 ± 0.21^a^	−1.26 ± 0.40^a^	−0.99 ± 0.32^a^	−1.24 ± 0.30^a^
Crumb b	26.91 ± 0.00^b^	29.74 ± 0.18^b^	29.19 ± 0.17^b^	27.26 ± 0.32^b^
Crumb whiteness	64.15 ± 0.68^a^	61.91 ± 0.52^b^	62.10 ± 0.53^b^	65.33 ± 0.35^a^
Hardness (*N*)	5.27 ± 0.08^a^	4.85 ± 1.61^a^	5.30 ± 0.39^a^	3.42 ± 0.61^b^
Resilience	0.42 ± 0.04^a^	0.39 ± 0.01^a^	0.37 ± 0.02^a^	0.43 ± 0.03^a^
Cohesiveness	0.71 ± 0.08^a^	0.62 ± 0.01^a^	0.60 ± 0.02^a^	0.65 ± 0.01^a^
Springiness (mm)	7.24 ± 0.30^a^	7.05 ± 0.00^a^	7.03 ± 0.11^a^	6.94 ± 0.25^a^
Gumminess (*N*)	3.72 ± 0.33^a^	2.99 ± 0.93^a^	3.15 ± 0.33^a^	2.23 ± 0.35^a^
Chewiness (mj)	26.40 ± 3.54^a^	20.65 ± 6.43^a^	21.70 ± 2.55^a^	15.20 ± 2.97^b^

Note

Control = bread without MAase and BW; MAase = bread containing free MAase; BW = bread containing BW; BW‐MAase = bread containing encapsulated MAase into BW. The values are expressed as Mean ± Standard deviation. Means with superscripts of different letters are significantly different (*p* < .05).

### 3.7 Textural parameters of gluten‐free breads

Texture profile analysis was done on gluten‐free breads and the results are shown in Table 3 including hardness, resilience, cohesiveness, springiness, gumminess, and chewiness. The mechanical features of bread are a function of the crumb structure and in particular, crumb hardness has been studied widely due to its high correlation to sensory evaluations. As can be seen in Table 3, although there were no significant differences in hardness and chewiness of control, and bread with MAase and bread with BW (*p* > .05), breads with BW‐MAase showed the lowest hardness and chewiness than other breads.

### 3.8 Microstructure of gluten‐free breads

**Figure 3:**
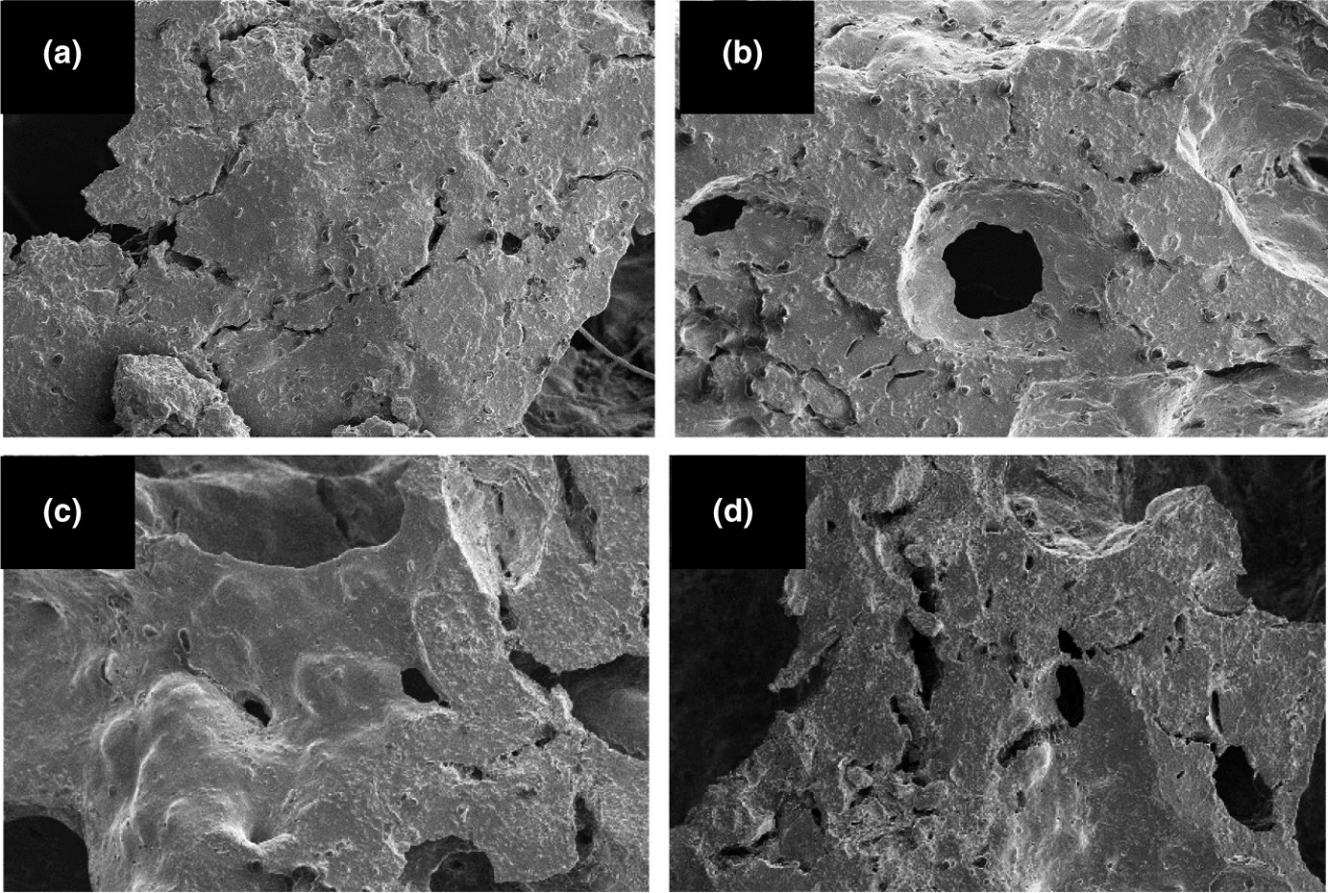
*SEM* micrographs of crumb of different gluten‐free breads. (a) Control, without MAase and BW; (b) MAase, containing free MAase; (c) BW, containing BW; (d) BW‐MAase, containing encapsulated MAase into BW. Magnification was 100×

The *SEM* was also used to investigate cellular structure of bread crumb of different gluten‐free breads (Figure 3). In general, breads with formulations of control and BW resulted in a rough structure and lower gas cells than other breads. Breads made from encapsulated MAase into BW resulted in more gas cells and more aerated structure in comparison with other breads, in other hand the breads having such structures and morphologies resulted in a better quality and retard in staling. Based on the results given in Tables 2 and 3, particularly lower weight loss, higher volume index, darker crust, whiter crumb, less hardness in crumb and also more aerated microstructure, one can conclude that breads made from the encapsulated MAase with BW exhibited a better quality in comparison with other formulation breads.

### 3.9 Gluten‐free bread staling

**Figure 4:**
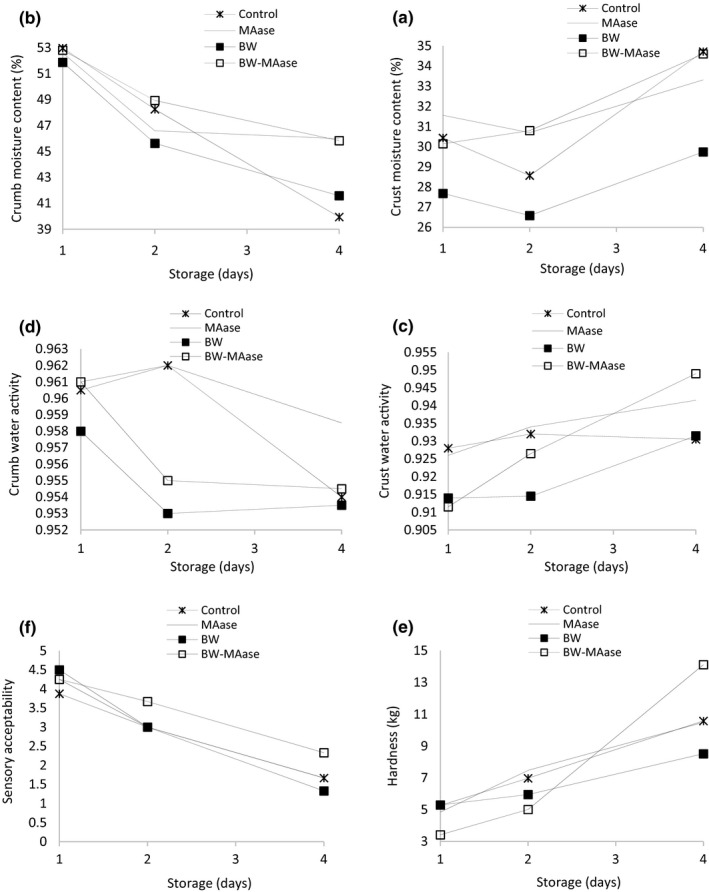
Changes in moisture content of crust (a) and crumb (b), water activity of crust (c) and crumb (d), hardness (e), and sensorial acceptability (f) of gluten‐free breads after 1, 2, and 4 days from baking

The variation of moisture content, water activity (a_w_) for the crust and crumb, hardness and sensorial acceptability of different gluten‐free breads during four days of storage are presented in Figure 4. Water is the main plasticizer in foods. Plasticizers make foods softer via embedding between polymer chains, reducing attraction forces between them and reducing T_g_. The plasticizing effect has a great importance in shelf life and sensorial properties of foods (Furlán et al., 2015). When bread started cooling, the difference in vapor pressure between crumb and crust resulted in the migration of moisture from crumb to crust of loaf, which ultimately leading to a decrease in moisture content of crumb and an increase in moisture content of the crust (Sabanis & Tzia, 2011). According to Figure 4a, b, c, and d, breads with BW‐MAase and MAase formulations had higher moisture content and water activity in crust and crumb than control and BW bread, keeping the structure of crumb softer than other breads after 4 days of baking.

Hardness for all bread samples increased with an increase in the storage time. Among bread samples, the bread with BW‐MAase formulation had softer crumb structure than other breads after 2 days of storage time (*p* < .05), which could be attributed to its higher moisture content in comparison with the other bread samples. However, the hardness of BW‐MAase bread on the fourth day of storage reached the highest in comparison with other breads, and still, its sensorial acceptability is higher in both 2 and 4 days after baking.

## 4 CONCLUSION

In this study, MAase, which is a kind of antistaling enzyme, was encapsulated into beeswax and its application on gluten‐free batter and bread quality and staling properties was determined. Results showed that this encapsulation technique had relatively good enzymatic efficiency, and its application in gluten‐free bread could lead to maintaining more water content, higher volume index, darker crust, and whiter crumb and reduce the hardness of baked product. However, this encapsulation system increases hardness of gluten‐free bread after 4 days of baking, and prepared bread still has a good sensorial acceptability. Therefore, application of beeswax in enzyme encapsulation for the baking industry can be considered as a good wall material.

## INFORMED CONSENT

Written informed consent was obtained from all study participants.

## CONFLICT OF INTEREST

The authors declare that they do not have any conflict of interest.

## ETHICAL APPROVAL

This study does not involve any human or animal testing.
